# Long term outcomes after COVID-19 in patients with schizophrenia: a historical cohort study in a health maintenance organization

**DOI:** 10.1007/s00127-025-02860-0

**Published:** 2025-03-03

**Authors:** Gashaw Getaneh Dagnaw, Ora Paltiel, Asher Shafrir

**Affiliations:** 1 College of Veterinary Medicine and Animal Sciences, Department of Epidemiology and Public Health, University of Gondar, Gondar, Ethiopia; 2 Braun School of Public Health and Community Medicine, Jerusalem, Israel; 3 Department of Haematology, Faculty of Medicine, Hadassah-Hebrew University, Jerusalem, Israel; 4 Department of Gastroenterology, Faculty of Medicine, Hadassah-Hebrew University, Jerusalem, Israel

**Keywords:** COVID-19, Hospitalization, Mortality, SARS-CoV-2, Schizophrenia

## Abstract

**Background:**

Severe mental illness may affect health behaviors and outcomes during pandemics. Few studies have assessed whether people living with schizophrenia spectrum disorders (SSD) experienced adverse COVID-19 outcomes.

**Methods:**

In a population-based historical cohort study comprising members of a health maintenance organization, we included 1273 patients with SSD and 12,730 age- and sex-matched controls tested for SARS-CoV-2 between March 2020 and May 2022. We assessed the association between schizophrenia and hospitalization, hospital length-of-stay, 30-day, and one-year mortality, constructing multiple linear regression and logistic regression models adjusting for sociodemographic factors, BMI, smoking, number of comorbidities, and vaccinations. We also assessed whether vaccination modified the association between schizophrenia and mortality.

**Results:**

Among patients with SSD, 477 (37.5%) had a positive test, compared to 6203 (48.7%) in the comparison group. patients with SSD were at increased risk of hospitalization (adjusted odds ratio (OR_adj_) 3.44, 95% confidence interval (CI): 2.88–4.11, *p* < 0.001); longer length-of-stay (β = 1.20, *p* < 0.001); increased 30-day (OR_adj_ 9.07, 95%CI 3.11–26.44); and one-year mortality (OR_adj_ 6.27, 95%CI: 2.73–14.39). Further adjustment for vaccination altered the OR for 30-day mortality (OR_adj_ 4.54, 95%CI: 1.54–13.38). Additionally, the association between schizophrenia and 30-day mortality was attenuated in strata of vaccinated (OR 4.79, 95%CI: 0.82–28.13, *p* = 0.082), vs. unvaccinated individuals (OR 7.53, 95%CI 2.19–25.92, *p* = 0.001), respectively.

**Conclusions:**

In our cohort, patients with SSD experienced a significantly higher rate of hospitalization, length of stay, and mortality following a positive SARS-CoV-2 test, even after adjusting for important prognostic factors. COVID-19 vaccination modified these risks.

**Supplementary Information:**

The online version contains supplementary material available at 10.1007/s00127-025-02860-0.

## Introduction

Factors known to be associated with adverse outcomes among people infected with SARS-CoV-2 include male sex, older age, and pre-existing medical conditions such as diabetes mellitus, cardiovascular diseases (CVD), obesity, and chronic obstructive pulmonary disease (COPD) [1, 2]. People living with schizophrenia spectrum disorders (SSD) were reported to have a decreased lifespan in general [3] and experience an increased risk of SARS-CoV-2 infections compared to the general population [4, 5] in the early waves of the pandemic. With the emergence of the highly infectious Omicron variant of SARS-CoV-2 in November 2021, all segments of the population were deemed susceptible to infection, but variability in the risk of complications remained [6].

Several studies reported that COVID-19-related hospitalizations and mortality were more frequent in patients with SSD compared to the general population [5, 7–11]. A systematic review and meta-analysis with pooled results of COVID-19-caused mortality showed a 2.22-fold higher risk of death due to COVID-19 in those with schizophrenia compared with those without schizophrenia [10]. A study conducted in France among patients admitted to acute care hospitals found higher mortality in patients with schizophrenia (26.7%) compared to patients without (8.7%) [12]. Similarly, a study conducted in Israel from March – October 2020 reported that patients with SSD experienced higher overall COVID-19 mortality and hospitalization in the first wave of the pandemic [13]. Most of the previous reports have attributed the higher incidence of mortality and hospitalization in patients with SSD to socioeconomic deprivation, poor healthcare access, and the presence of medical comorbidities [14]. Adverse outcomes among patients with SSD may also be attributed to a weakened immune system, under-utilization of vaccination and stigma (6,7 [15]). Additionally, patients with SSD have been reported to have poorer adherence to personal prevention practices, such as poor hygiene, low levels of awareness, and difficulty with social distancing [8]. Lastly, antipsychotic drugs were correlated with worse COVID-19 outcomes [16]. 

Most studies reporting the outcomes of patients with SSD were conducted during the early stages of the pandemic and did not encompass the major waves of the outbreak. While a number of studies in the general population have demonstrated an increased one-year mortality after infection by SARS-CoV-2 [17], limited information is available regarding whether this risk is enhanced in patients with SSD.

We hypothesized that schizophrenia independently increases the probability of adverse COVID-19 outcomes due to its pathophysiological link with immunity, and with cardio-metabolic risk factors [15, 17] which are, in part, related to antipsychotic medications. Furthermore, the high prevalence of smoking in this population, as well as to issues of access of care, which persist even in countries with universal health coverage, may pose additional risks to patients with SSD when faced with SARS-CoV-2 infection.

## Materials and methods

### Study population and data sources

This study utilized the databases of the Meuhedet Health Maintenance Organization (HMO). Meuhedet HMO is one of Israel’s four national health insurance providers. It provides health services for approximately 1.3 million people. We conducted a population-based historical cohort study using the database.

The Meuhedet HMO offers comprehensive and integrated medical services, including primary care, emergency services, hospitalization, maternity care, dental care, and mental health services for a diverse membership of Israeli residents [18]. Its database is continuously updated with real-time data collected from medical service centers and include clinical and laboratory data. The database is linked with the National Population Registry for vital status and all hospitalizations are incorporated regardless of where they take place in the country. Additionally, all SARS-CoV2 test results were reported to the HMOs.

During the study period, 1.07 million members of the Meuhedet HMO had a SARS-CoV-2 PCR test. Among these, nearly 700,000 were adults, and 1273 had a recorded diagnosis of schizophrenia. The study population consisted of people living with schizophrenia, who were diagnosed prior to infection with SARS-CoV-2, and a randomly-chosen sample of adults aged 18 years and older who underwent a SARS-CoV-2 test and no diagnosis of schizophrenia. Individuals who were under the age of 18 years were excluded. Patients with a recorded diagnosis of schizophrenia in the electronic medical record (EMR) were considered to have schizophrenia. (See supplement A for the list of ICD – codes that were used). For each patient with schizophrenia, we randomly chose 10 controls, matched on age and gender. Of these, we recorded results and dates of SARS-CoV-2 tests by polymerase chain reaction (PCR) performed between March 2020 to May 2022, available free of charge via the HMO or government testing centers. Vaccination status recorded by date. Patients were considered vaccinated 7 days after the second vaccination using either the Pfizer (Pfizer-BioNTech) or Moderna (Spikevax) mRNA vaccine.

For eligible individuals we extracted data from the electronic medical record including: sociodemographic information such as age, sex, sector, and socioeconomic status (SES) smoking status, body mass index (BMI), and health-related data such as medical comorbidities, SARS-CoV-2 test results, hospitalizations, intensive care unit (ICU) admission, length of stay in the hospital, schizophrenia, vaccination, death and date of death.

### Definition of study variables

The main outcome variables included mortality, hospitalization, and length of hospitalization. Mortality was defined as death from all causes within 30 days or one-year after testing positive for SARS-CoV-2 by PCR. Hospitalization referred to hospital admission occurring withing two months after the first SARS-CoV-2 positive test.

Length of hospitalization was defined as the number of days from admission to discharge or death at the hospital after the patient has been hospitalized with COVID-19.

Age was extracted as a continuous variable and then reclassified into categories: <50, 50–59, 60–69, 70–79 years of age, and 80 years old and above. Sex was based on reported male or female gender. The HMO serves various sectors in the population; these are characterized based on the religious affiliation and/or degree of religious observance of the majority attending the clinic, and categorized into non- ultra-Orthodox Jews, Arabs, and Ultra-orthodox Jews. Socioeconomic status (SES) determination was based on an ordinal scale from 1 to 10 (and further grouped into low [1–4], medium [5–7], and high [8–10] socioeconomic levels) according to a scale based on the HMO member’s neighborhood’s income, education, and consumer activity. (POINTS Location Intelligence Company Ramat Gan).

Comorbidities included medical conditions: chronic obstructive pulmonary disease (COPD), hypertension, heart failure, rheumatic heart disease, hypothyroidism, asthma, type-2 diabetes, depression, atrial fibrillation, cancer, stroke, chronic renal failure, and dementia, recorded in the EMR at any point prior to SARS-CoV-2 testing. We determined the total number of conditions present and categorized them into three groups (0, 1, 2, ≥ 3 conditions recorded). Smoking status referred to active smokers based on the clinical records and was collected as a dichotomous variable. We determined if participants had received any vaccination and also the number of vaccinations. Body mass index (BMI weight (kg) per height (m^2^)) was assessed as a continuous variable.

### Data analysis

Categorical variables were summarized as counts and proportions, and continuous variables as means with standard deviations (SDs). Categorical outcomes such as hospitalization, ICU admission, 30-day mortality, and 1-year mortality) were compared using a chi-square test. Continuous variables such as length of hospitalization were compared using the Mann-Whitney U test. To assess the association between schizophrenia and dichotomous study outcomes, crude, multivariable-adjusted (for socio-demographic variables, BMI, smoking, and number of comorbidities) and lastly fully-adjusted (all of the above plus vaccinations), logistic and linear regression models were constructed. Effect of different variables was reported as odd ratios (OR) and 95% confidence interval (CI).

Since length of hospitalization was not normally distributed but rather right-skewed, we used a log-transformation to assess this outcome, performing multiple linear regression adjusted for the covariates mentioned above.

We explored potential effect modification of the relationship between schizophrenia and mortality by vaccination in age-adjusted models stratified by vaccination status. To formally assess this interaction, an interaction term of SSD by vaccination status use was introduced into the multivariable logistic regression analysis of mortality.

Missing values were omitted from the analysis, ensuring complete cases were used for statistical modelling. Odds ratio (OR) with 95% CI and β-coefficient with standard error (SE) were reported and p-values less than 0.05 were considered statistically significant. All statistical analyses were performed by R 4.3.0 software, and packages such as “Dplyr”, “Publish”, “tableone”, “stats”, “tidyr”, “Car”, “lubridate” and “ggplot2” were used. The study was performed according to strengthening the reporting of observational studies in epidemiology (STROBE) guidelines (Supplementary Check List 1).

The study was approved by the research ethics committee and internal review board of Meuhedet HMO (02-24-08-20, 2 September 2020).

## Results

**Fig. 1:**
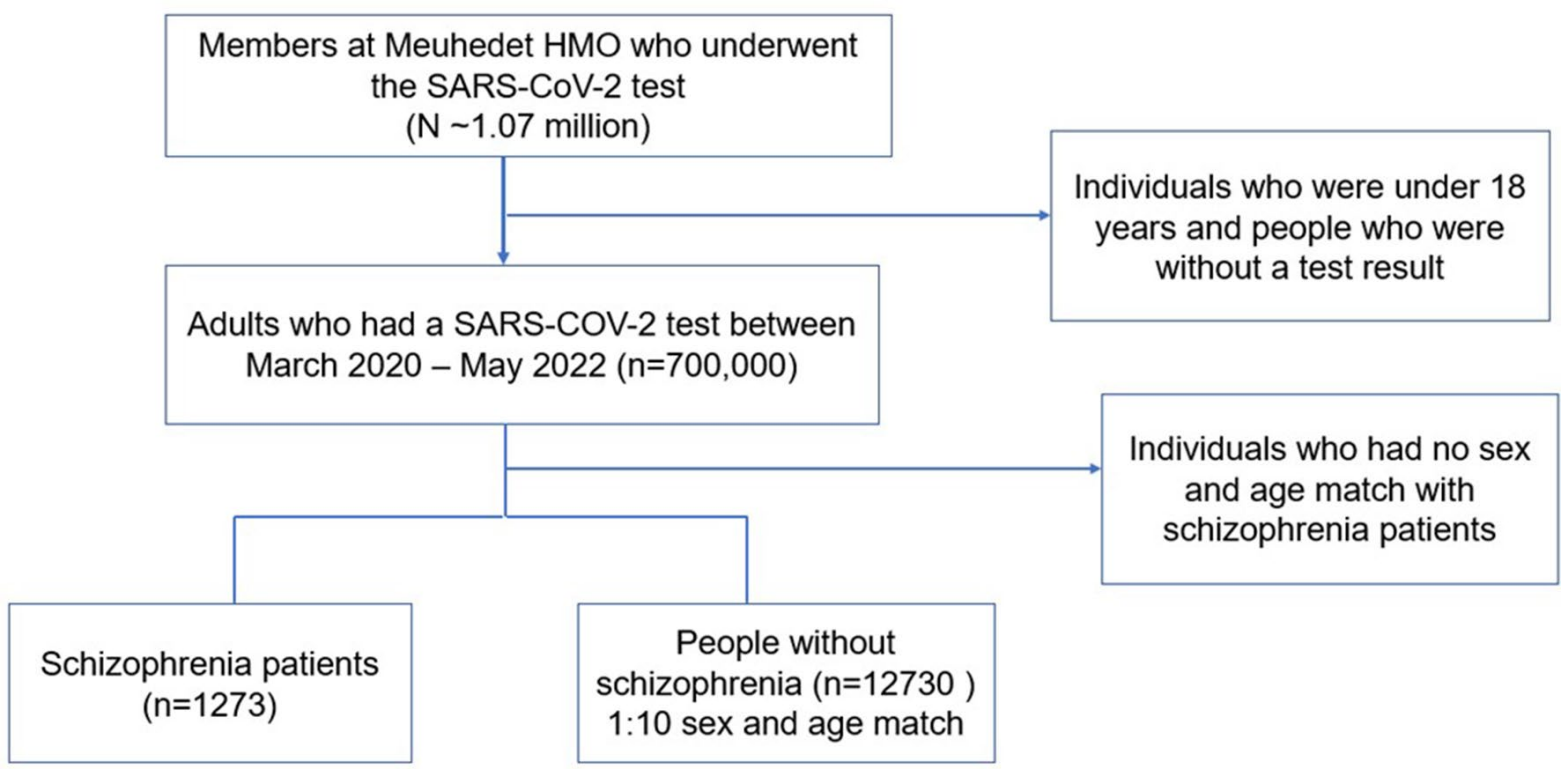
Flowchart of 14,003 individuals included in this study

A total of 14,003 individuals (≥ 18 years of age) who had a SARS-CoV-2 test between March 2020 – May 2022 in the Meuhedet HMO were included in the final study population (See Fig. 1 for the flow diagram of study participants). Of these, 1273 were diagnosed with schizophrenia and these were matched by age and sex to 12,730 individuals without schizophrenia. Sociodemographic characteristics, smoking status, and BMI of the study population are presented in Table 1. The average age was 48.05 years (± 14.92) and 2.2% of the participants were aged 80 years and above. Males comprised 58.8% of the study population. Approximately 26.4% of individuals diagnosed with schizophrenia belonged to the Ultra-Orthodox Jewish subpopulation, compared to 21.6% of the control group (p-value < 0.001), 9.1% of people with schizophrenia and 16.5% of the controls belonged to the Arab minority population (p-value < 0.001). Among patients with SSD, 30.3% were smokers, compared to only 17.4% of controls (p-value < 0.001). BMI for patients with SSD was 29 kg/m^2^ ± 6.2, compared to 27.3 kg/m2 ± 5.3 in the control group. A higher percentage of individuals with schizophrenia (42.1%) were from a low socioeconomic position, compared to 36.1% of the control group.

**Table 1:** Descriptive sociodemographic characteristics of the study populations comparing individuals with and without schizophrenia

Categories	Schizophrenia (%) (*n* = 1273)	Control (%) (*n* = 12730)	Total (%) (*n* = 14,003)	*p*-value
Sex				1.00
Female	525 (41.2)	5,250 (41.2)	5,775 (41.2)	
Male	748 (58.8)	7,480 (58.8)	8,228 (58.8)	
Age (mean ± SD)	48.05 ± 14.92	48.05 ± 14.93		1.00
Age (%)				1.00
< 50	705 (55.4)	7050 (55.4)	7,755 (55.4)	
50–59	288 (22.6)	2880 (22.6)	3,168 (22.6)	
60–69	174 (13.7)	1740 (13.7)	1,914 (13.7)	
70–79	78 (6.1)	780 (6.1)	858 (6.1)	
80+	28 (2.2)	280 (2.2)	308 (2.2)	
Sector (%)				< 0.001
Arab	116 (9.1)	2097 (16.5)	8,228 (58.8)	
Non-Haredi Jews	821 (64.5)	7867 (61.8)	2,213 (15.8)	
Ultra-Orthodox Jews	336 (26.4)	2752 (21.6)	8,688 (62.0)	
Missing	0 (0.0)	14 (0.1)	14 (0.1)	
SES (%)				< 0.001
1–4	536 (42.1)	4591 (36.1)	5,127 (36.6)	
5–7	591 (46.4)	5776 (45.4)	6,367 (45.5)	
8–10	132 (10.4)	2162 (17.0)	2,294 (16.4)	
Missing	14 (1.1)	201 (1.5)	215 (1.5)	
BMI (mean ± SD, kg/m²)	29 ± 6.2	27.3 ± 5.3		< 0.001
Smoking - yes (%)	386 (30.3)	2,219 (17.4)	2,605 (18.6)	< 0.001

Comorbidities were more frequently observed in individuals with schizophrenia, the most common being depression (56.4% vs. 13/%, p = value < 0.001), type 2 diabetes mellitus (DM) (19.6% vs. 9.7%, p-value < 0.001), hypothyroidism (13.4% vs. 7.5%, p-value < 0.001), COPD (12.5% vs. 5%, p-value < 0.001), hypertension (10.1% vs.7.5%, p-value < 0.001). Among individuals with schizophrenia, 26.9% had no comorbidities, 37.6% had one, 18.1% had two, and 17.4% had three or more comorbidities. Additional comparisons are presented in Table 2. The association between individual medical comorbidities with COVID-19-related hospitalizations and mortality is presented in Table S1 and Table S2.

**Table 2:** Pre-existing medical conditions and vaccination status in individuals with schizophrenia compared to those without schizophrenia

Characteristics	Patients with SSD (%) (*n* = 1273)	Without SSD (%) (*n* = 12730)	Total (%) (*n* = 14,003)	*p*-value
Depression (%)	718 (56.4)	1,672 (13.1)	2,390 (17.1)	< 0.001
Diabetes type 2 (%)	249 (19.6)	1,236 (9.7)	1,485 (10.6)	< 0.001
Hypothyroidism (%)	171 (13.4)	950 (7.5)	1,121 (8.0)	< 0.001
COPD (%)	159 (12.5)	632 (5.0)	791 (5.6)	< 0.001
Hypertension (%)	129 (10.1)	951 (7.5)	1,080 (7.7)	0.001
Asthma (%)	64 (5.0)	548 (4.3)	612 (4.4)	0.26
Ischemic heart disease %)	61 (4.8)	526 (4.1)	587 (4.2)	0.29
Heart failure (%)	40 (3.1)	174 (1.4)	214 (1.5)	< 0.001
Cancer (%)	73 (5.7)	888 (7.0)	961 (6.9)	0.11
Number of comorbidities				
0	343 (26.9)	7,965 (62.6)	8,308 (59.3)	< 0.001
1	479 (37.6)	2,885 (22.7)	3,364 (24.0)	< 0.001
2	230 (18.1)	1,081 (8.5)	1,311 (9.4)	< 0.001
≥ 3	221 (17.4)	799 (6.3)	1,020 (7.3)	< 0.001
Vaccination status				
Vaccination 1 (%)	1015 (80.0)	10,665 (84.0)	11,680 (83.4)	0.001
Vaccination 2 (%)	942 (74.0)	9,637 (75.7)	10,579 (75.5)	0.19
Vaccination 3 (%)	752 (59.1)	7,630 (59.9)	8,382 (59.9)	0.57

SSD = schizophrenia spectrum disorders

A total of 11,680 (83.4%) of the study population were vaccinated within the first round of vaccination, among these, 1015 (80.0%) were patients with SSD, a significantly lower percentage than among the controls (84%, *p* < 0.001). However, there was no statistically significantly difference for the second and third rounds (*p* = 0.188 and p-value = 0.569, respectively) (Table 2).

### COVID-19-related outcomes in people with and without schizophrenia

**Fig. 2:**
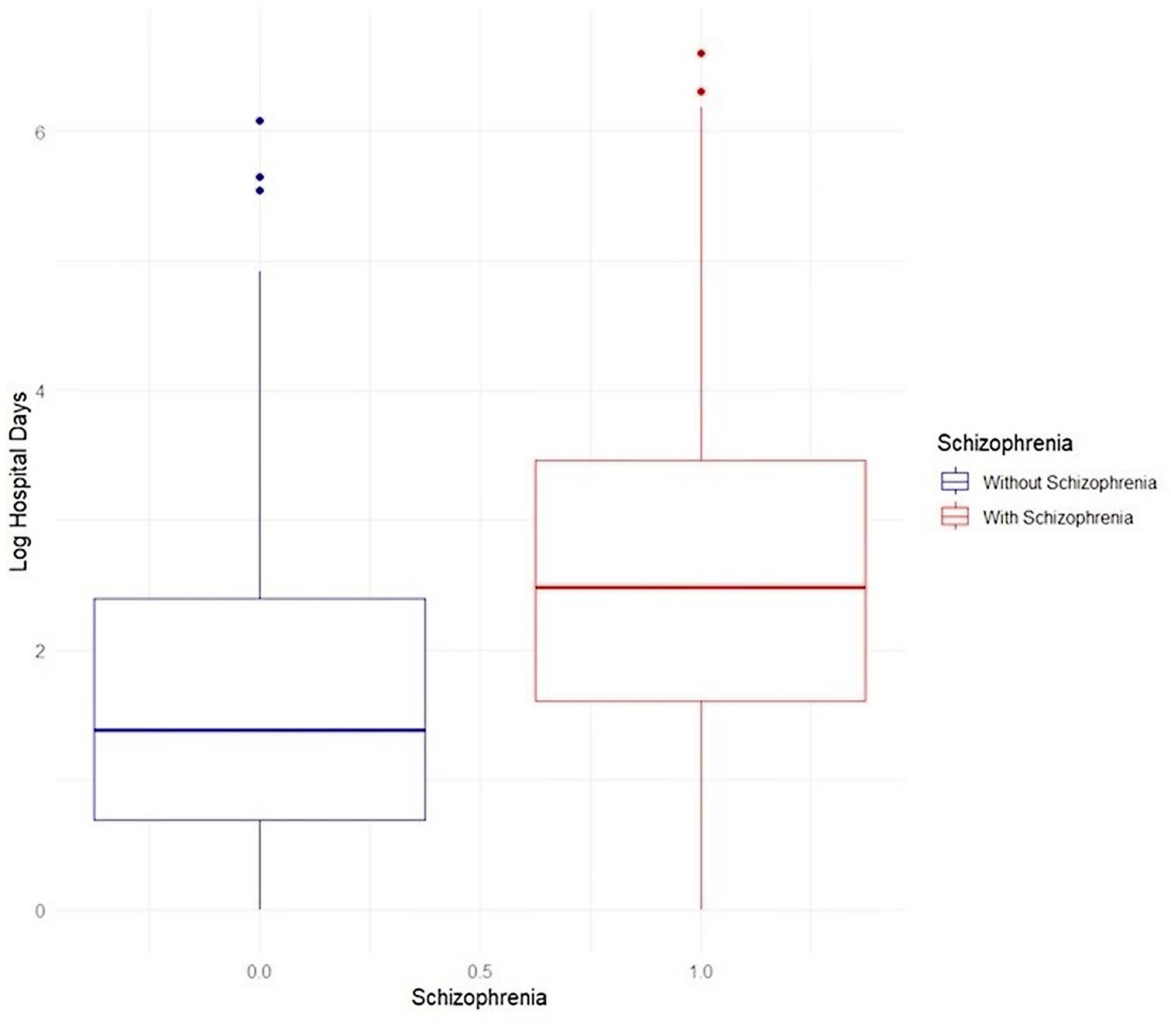
Box Plot showing the length of hospitalization of people with (blue box) and without schizophrenia (red box)

Table 3 illustrates SARS-CoV-2 infection sequelae. Compared to controls, patients with SSD were less likely to have a positive test for SARS-CoV-2 (37.5% vs. 48.7%, p-value < 0.001). Among patients with a positive SARS-CoV-2 test hospitalization, length of hospitalization, and all-cause 30-day and one-year mortality were more frequently observed among schizophrenia patients compared to healthy controls (all *p* < 0.001). Among patients with SSD who tested positive, 230 (48.2%) were hospitalized, and 54 (11.3%) were hospitalized for two or more days. Figure 2 illustrates that the median length of hospitalization for patients with SSD was more than two days compared to less than two days in the control group. Among patients with SSD following COVID-19, one-year mortality was 7.8%, compared to 1.5% in the comparison population (p-value < 0.001). Only three (0.6%) schizophrenia patients were admitted to an ICU (*p* = 0.307, Table 3), and hence, we did not further analyze this outcome further in multivariable models.

**Table 3:** SARS-CoV-2 infections, hospitalization, length of hospitalization, ICU admission, and all-cause mortality among SARS-CoV-2 test positives in people living with schizophrenia compared to controls

Vaccination and COVID-19 outcomes	Schizophrenia (*n* = 1273)	Without schizophrenia (*n* = 12730)	*p*-value
COVID-19 (%)	477 (37.5)	6203 (48.7)	< 0.001
*Hospitalization (%)	230 (48.2)	686 (5.4)	< 0.001
*Length of hospitalization (%)			< 0.001
0 day	247 (51.8)	5517 (88.9)	
1 day	176 (36.9)	542 (8.7)	
≥ 2 days	54 (11.3)	144 (2.3)	
*ICU admission (%)	3 (0.6)	12 (0.2)	0.31
*All-cause 30-day mortality (%)	14 (2.9)	37 (0.6)	< 0.001
*All-cause 1-year mortality (%)	37 (7.8)	91 (1.5)	< 0.001

* Among individuals with positive SARS-CoV-2 tests

Schizophrenia was significantly associated with hospitalization (OR = 5.18, 95%CI = 3.90–6.89, *p* < 0.001) (Table 4). In a multivariable-adjusted logistic regression model controlling for sex, age, sector, SES, BMI, smoking, and the number of medical comorbidities, schizophrenia was independently associated with increased hospitalization (OR = 4.49, 95%CI = 3.29–6.13, *p* < 0.001) (Table 4). Other factors related to hospitalization included multiple comorbidities, advanced age, smoking, and SES. The association between schizophrenia and COVID-19 hospitalization remained significant but was attenuated after adding vaccination to the model (OR = 3.80, 95%CI = 2.70–5.34, *p* < 0.001) (Table S6), even though vaccination had a dose-responsive protective effect.

Schizophrenia patients experienced a longer length-of-stay after being admitted to the hospital following SARS-CoV-2 positive test (β = 1.24, *p* = 0.003) (Table 4). After multivariable adjustment for sex, age, sector, SES, BMI, smoking, and number of medical comorbidities, a multiple linear regression analysis showed a statistically significant association between schizophrenia and log-transformed number of hospital days (β = 1.2, *p* < 0.001) (Table 4). This association remained significant when performing a Poisson regression. After further adjustment for vaccination, the association between schizophrenia and length of hospitalization maintained its statistical significance (β = 1.18, *p* < 0.001) (Table 4). Furthermore, vaccination had a protective effect on the length of hospitalization (Table S6).

Individuals with schizophrenia had significantly higher odds of 30-day mortality compared to those without schizophrenia (OR = 7.54, 95%CI: 3.15–18.07, *p* < 0.001) (Table 4). The association was strengthened (OR = 9.07, 95%CI: 3.11–26.44, *p* < 0.001) after adjustment for sex, sector, SES, age, BMI, smoking, and the number of comorbidities (Table 4). The association between schizophrenia and 30-day mortality was attenuated after further including vaccination (OR = 4.54, 95%CI: 1.54–13.38, *p* = 0.006) (Table 4). Advanced age, multiple comorbidities and smoking, as well as sector and SES were other factors related to 30-day mortality (Table S5).

Schizophrenia was significantly associated with increased one-year mortality (OR = 7.78, 95%CI: 4.00–15.15, *p* < 0.001) (Table 4). This finding remained statistically significant in a multivariate logistic regression, adjusting for sex, sector, SES, age, BMI, smoking, and number of comorbidities (OR = 6.27, 95%CI: 2.73–14.39, *p* < 0.001) (Table 4). The association was maintained after the effect of vaccination was taken into consideration (OR = 4.98, 95%CI: 2.23–11.15, *p* < 0.001) (Table 4). Vaccination had a protective effect (Table S6).

Formal testing using multivariate logistic regression confirmed the presence of a statistically significant interaction between vaccination status and SSD (*P* < 0.001).

In stratified age-adjusted models, among individuals who had received vaccination, the effect of schizophrenia on 30-day mortality was attenuated (OR = 4.79, 95%CI = 0.82–28.13, *p* = 0.082), whereas, in unvaccinated individuals, schizophrenia had a statistically significant effect on 30-day mortality (OR = 7.53, 95%CI = 2.19–25.95, *p* = 0.001) (Table 5). However, vaccination did not appear to attenuate the link between schizophrenia and one-year mortality. The association of schizophrenia on one-year mortality was evident both in vaccinated (OR = 10.24, 95%CI = 3.25–32.31, *p* < 0.001) and unvaccinated individuals (OR = 5.76, 95%CI = 2.13–15.60, *p* = 0.001) (Table 5).

**Table 4:** Crude and multivariable-adjusted associations between schizophrenia and COVID-19-related hospitalization, length of hospitalization and all-cause mortality^#^

Outcome variable	Schizophrenia					
	Crude OR (95%CI)^a^	*P*-value	Adjusted OR (95%CI)^b^	*P*-value	Adjusted OR (95%CI)^c^	*P*-value
Hospitalization	5.18 (3.90–6.89)	< 0.001	4.49 (3.29–6.13)	< 0.001	3.80 (2.70–5.34)	< 0.001
All-cause 30-day mortality	7.54 (3.15–18.07)	< 0.001	9.07 (3.11–26.44)	< 0.001	4.54 (1.54–13.38)	0.006
All-cause 1-year mortality	7.78 (4.00–15.15)	< 0.001	6.27 (2.73–14.39)	< 0.001	4.98 (2.23–11.15)	< 0.001
Length of hospitalization (log-transformed)	β-coefficient (SE)^a^	p-value	β-coefficient (SE)b	p-value	β-coefficient (SE)c	p-value
	1.24 (0.423)	< 0.001	1.20 (0.063)	< 0.001	1.18 (0.063)	< 0.001
	-1.06 (0.226) *	< 0.001	-6.80 (0.107) *	< 0.001	-6.53 (0.113) *	< 0.001

a = unadjusted odd ratio (crude odd ratio) and β-coefficient, b = adjusted for sex, sector, SES, age, BMI, smoking, and for the number of comorbidities among SARS-CoV-2 positive individuals, c = adjusted including vaccination, SE = standard error. *= intercept for log-transformed length of hospitalization

*# = analysis by multiple logistic or linear regression as appropriate*

**Table 5:** Logistic regression model showing age-adjusted analysis of the association between schizophrenia and mortality among SARS-CoV-2-positive individuals stratified by vaccination status

Stratum	Schizophrenia		
	30-day mortality		
	OR	95%CI	*p*-value
Vaccinated (*N* = 11680)	4.79	0.82–28.13	0.082
Non-vaccinated (*N* = 2323)	7.53	2.19–25.92	0.001
One-year mortality			
Vaccinated (*N* = 11680)	10.24	3.25–32.31	< 0.001
Non-vaccinated (*N* = 2323)	5.76	2.13–15.60	0.001

## Discussion

In this population-based historical cohort study, we investigated the association between schizophrenia with COVID-19 outcomes, namely hospitalizations, length of hospitalization, ICU admission, and all-cause 30-day and one-year mortality. We found that schizophrenia is significantly associated with increased odds of hospitalizations, length of hospitalization, and all-cause 30-day and one-year mortality.

We did not find higher infection rates of SARS-CoV-2 among patients with SSD, however, schizophrenia was associated with higher rates of hospitalization, indicating a more serious illness. This finding is in line with a previous report by Murphy et al. [14] from the USA which showed that individuals with severe mental illness including schizophrenia were hospitalized more often compared to those without. Two previous studies from Israel showed that schizophrenia patients were more often hospitalized than people without schizophrenia [9, 19]. Multiple factors contribute to the increased risk of hospitalizations in schizophrenia patients. According to Tzur et al. [9], schizophrenia patients were under-vaccinated compared to the general population, in a study conducted early in the pandemic.

We noted that there was a significant disparity in vaccination between people with and without schizophrenia in receiving the first vaccination during the early stage of the pandemic. However, in the second and third vaccination rounds (among those vaccinated), we observed that the difference was insignificant. Our analysis showed that vaccination had a protective effect, nevertheless, the association between schizophrenia and hospitalization remained strong. The strong association between schizophrenia and hospitalizations may suggest a contribution of altered immunity due to schizophrenia but may also suggest heightened concern by physicians treating these patients regarding their prognosis either because of schizophrenia or because of comorbidities.

Individuals with schizophrenia experienced extended hospitalization after COVID-19 admission. This is similar to findings in other studies [20, 21]. Prolonged hospitalization can be due to lack of family support, financial constraints, and the patient’s underlying health conditions according to pre-pandemic studies [21]. Our study results consistently shows that schizophrenia is significantly associated with increased length of hospitalizations even after accounting for other risk factors.

Similar to other studies, we chose dichotomous endpoints such as 30 day and 1 year mortality, In our study schizophrenia was associated with 30-day and one-year mortality in the setting of COVID-19 independently of other risk factors. Several studies found similar finding regarding 30-day mortality. Two population-based studies from France [22] and the United States [8] reported higher odds of mortality among schizophrenia patients compared with the general population. Another study from the USA by Murphy et al. [14] found a higher risk of 30-day mortality after a COVID-19 diagnosis in people with severe mental illness, including schizophrenia. Similarly, higher 45-day mortality among schizophrenia was reported by Nemani et al. [23]. The present findings are consistent with a study conducted by Tzur et al. [13] in Israel, which also reported higher rates of overall mortality among schizophrenia patients. In contrast, data regarding the long-term sequelae on COVID-19 in patients with SSD are scarce and our findings regarding one-year mortality are novel. Various factors have been proposed to explain the higher mortality rates among schizophrenia patients, among these is the presence of multiple medical conditions that make them more susceptible to death. While many of the risk factors were more common in patients with SSD, after controlling for these, schizophrenia was significantly associated with 30-day mortality. It should be noted that there are shared genes between SSD and metabolic syndrome which might influence COVID outcomes [24].

Most published studies interpreted the higher risk of mortality in schizophrenia patients as associated with barriers to the health system, stigmatization, and race. While these factors deserve serious consideration, it is important to consider potential effects of the biology of the disease itself. Previous research has indicated that schizophrenia is associated with genes that encode histocompatibility complex A that controls the immune response [25, 26], possibly causing schizophrenia patients’ diminished ability to fight SARS-CoV-2. In addition, imbalanced T-cell responses [27, 28], and inflammatory cytokine signalling disruptions are consistently observed in schizophrenia patients [29], which could contribute to severe COVID-19 outcomes. Furthermore, the Israeli health system provides universal coverage and access to all residents, potentially diminishing the effects of poor access.

Vaccination attenuated the effect of schizophrenia on 30-day mortality in individuals. This suggests that vaccination provides a protective effect against COVID-19-related 30-day mortality in individuals with schizophrenia, highlighting its importance in improving SARS-CoV-2-related outcomes, even among the most vulnerable. This effect of schizophrenia was not seen regarding one-year mortality. It is important to note that the outcome measured was all-cause mortality and therefore one year mortality may be unrelated to SARS-CoV-2 infection. In line with our findings, a study from Israel reported that vaccinated individuals with schizophrenia continue to have an elevated risk of adverse COVID-19 outcomes including mortality [19]. Another study reported that individuals with severe mental illnesses, including schizophrenia had a higher COVID-19 mortality even after vaccination [30]. This might be related to the use of antipsychotic medications, including commonly used medications like clozapine, which are used in the management of schizophrenia. For instance, long-term clozapine use has been linked to immunodeficiency risk [31].

Similar to other studies, age, low SES, comorbidities, increased the risk of mortality and longer hospitalization [2, 14, 22, 32]. It is known that patients with SSD frequently are found in lower socioeconomic positions compared to healthy controls [33].

This study has several strengths. The extended follow-up time of one year allowed us to ensure that most patients had reached all COVID-19 outcomes, enhanced the study’s statistical power and covered many of the major SARS-CoV-2 waves in Israel. Our study was population-based, in a country with universal health insurance, without financial barriers to testing, treatment and hospitalization for COVID-19 and included diverse patient populations including minorities such as Arabs, and Ultra-orthodox Jews with sufficient proportions, which allowed us to understand the impact of schizophrenia on COVID-19 outcomes in diverse sociodemographic and sociocultural groups. In addition to known risk factors for poor prognosis of COVID-19 outcomes, we considered additional covariates such as vaccination. In our study, controls were randomly selected from all members of the Meuhedet HMO. In addition, housing for patients with SSD is developed in Israel and there is no significant difference in the type of neighborhoods where patients with SSD live to healthy controls [34]. 

Notwithstanding, this study has several limitations. Having an assumed baseline prevalence of 0.3% [35], in the population, we would expect to have about 2100 cases of schizophrenia in this data set. Thus, we probably have an underestimate of schizophrenia in our study population. This suggested that people were underdiagnosed, possibly due to fear of stigmatization, social factors, or other reasons. This could result in differential misclassification that ultimately influences the outcome of the associations between schizophrenia and COVID-19 outcomes. In addition, we had limited data regarding additional psychiatric diagnoses i.e. bipolar disease and depression. Another limitation of our study was we did not have sufficient data on medications which may have impacted the COVID-19 outcomes. The antipsychotic drugs commonly used to manage symptoms of schizophrenia, may affect the immune system as well as being responsible for many of the cardiometabolic comorbidities and obesity that could potentially impact the outcomes of COVID-19. In addition, the number of individuals with schizophrenia admitted to the ICU was very limited, precluding the study of this outcome. The deaths ascertained were from any cause, and not necessarily due to COVID-19 and the dataset lacks information about the causes of death and reasons for hospitalization, which makes it difficult to establish a precise association between schizophrenia and COVID-19 outcomes.

## Conclusions

Patients with SSD are at increased COVID-19 hospitalizations, length of hospitalization, 30-day and one-year mortality. Individuals with schizophrenia should be prioritized in receiving preventive measures for infectious diseases for early detection and intervention.

## Electronic supplementary material

Below is the link to the electronic supplementary material.

Supplementary Material 1

## Data Availability

The datasets generated and/or analysed during the current study are not publicly available due to ethical restrictions on sharing our data set because data contain potentially identifying patient information but are available from the Meuhedet research center coordinator on reasonable request.

## Abbreviations

BMI: Body Mass Index
COPD: Chronic Obstructive Pulmonary Disease
COVID-19: Coronavirus Disease 2019
CVD: Cardiovascular Diseases
HMO: Health Maintenance Organization
ICD-9: International Classification of Diseases, Ninth Revision
ICU: Intensive Care Unit
OR: Odd Ratio
PCR: Polymerase Chain Reaction
SSD: Schizophrenia spectrum disorders
SARS-CoV-2: Severe Acute Respiratory Syndrome Coronavirus 2
SES: Socioeconomic status
