# Supplementary material for: Long term outcomes after COVID-19 in patients with schizophrenia: a historical cohort study in a health maintenance organization
Source: Soc Psychiatry Psychiatr Epidemiol. 2025 Mar 3;60(8):2013–22. doi: 10.1007/s00127-025-02860-0 (PMC12325438; doi:10.1007/s00127-025-02860-0)
Supplement: Supplementary file 1 — Supplementary Material 1 [file 127_2025_2860_MOESM1_ESM.docx]

**Appendix A: Supplementary data**

**Table S1.** Association of pre-existing comorbidities with all-cause death among schizophrenia patients compared to the control

| Characteristics | All-cause death | | |
| --- | --- | --- | --- |
|  | **OR** | **95%CI** | **P-value** |
| Schizophrenia | 1.98 | 1.28 - 3.08 | 0.002 |
| Hypertension | 2.85 | 1.90 - 4.27 | <0.001 |
| Depression | 1.67 | 1.13 - 2.47 | 0.01 |
| Heart Failure | 6.89 | 4.01 - 11.85 | <0.001 |
| Rheumatic Heart Disease | 4.64 | 0.35 - 62.42 | 0.247 |
| Atrial Fibrillation | 2.01 | 1.09 - 3.70 | 0.026 |
| Hypothyroidism | 0.77 | 0.44 - 1.34 | 0.348 |
| COPD | 3.33 | 2.17 - 5.13 | <0.001 |
| Asthma | 0.47 | 0.20 - 1.07 | 0.073 |
| Dementia | 6.21 | 2.06 - 18.68 | 0.001 |

**Table S2.** Association of pre-existing comorbidities with COVID-19-related hospitalization among individuals with SARS-CoV-2 positive tests

| Characteristics | Hospitalization | | |
| --- | --- | --- | --- |
|  | **OR** | **95%CI** | **P-value** |
| Schizophrenia | 2.97 | 2.47 - 3.57 | <0.001 |
| Hypertension | 2.17 | 1.78 - 2.64 | <0.001 |
| Depression | 1.44 | 1.22 - 1.71 | <0.001 |
| Heart Failure | 2.53 | 1.75 - 3.66 | <0.001 |
| Rheumatic Heart Disease | 1.47 | 0.13 - 16.98 | 0.758 |
| Atrial Fibrillation | 1.97 | 1.39 - 2.81 | 0.001 |
| Hypothyroidism | 1.18 | 0.95 - 1.48 | 0.142 |
| COPD | 2.07 | 1.65 - 2.59 | <0.001 |
| Asthma | 0.69 | 0.48 - 0.97 | 0.035 |
| Dementia | 3.47 | 1.50 - 8.06 | 0.004 |

**Table *S3*.** Multivariate logistic regression model showing the association between schizophrenia with COVID-19-related hospitalization adjusted for sex, sector, SES, age, BMI, smoking, and for the number of comorbidities among SARS-CoV-2 positive individuals

| Independent variables | Dependent variable | | |
| --- | --- | --- | --- |
|  | **Hospitalization** | | |
|  | **OR** | **95%CI** | **P-value** |
| Schizophrenia | 4.49 | 3.29 - 6.13 | < 0.001 |
| Male (vs female) | 1.34 | 1.13 - 1.59 | < 0.001 |
| Sector (non-Haredi Jews) | **Ref** |  |  |
| Arab | 1.12 | 0.87 - 1.43 | 0.390 |
| Ultra-Orthodox Jews | 0.72 | 0.56 - 0.91 | 0.006 |
| SES (1-4) | **Ref** |  |  |
| 5-7 | 0.79 | 0.65 - 0.97 | 0.021 |
| 8-10 | 0.62 | 0.47 - 0.82 | 0.001 |
| Age (<50) | **Ref** |  |  |
| 50-59 | 1.82 | 1.47 - 2.24 | < 0.001 |
| 60-69 | 2.32 | 1.82 - 2.95 | < 0.001 |
| 70-79 | 4.59 | 3.47 - 6.06 | < 0.001 |
| 80+ | 10.91 | 7.64 - 15.59 | < 0.001 |
| BMI (kg/m^2^) | 1.01 | 0.99 - 1.02 | 0.223 |
| Smoking (yes vs no) | 1.54 | 1.28 - 1.86 | < 0.001 |
| Comorbidities (0) | **Ref** |  |  |
| 1 | 1.20 | 0.96 - 1.49 | 0.117 |
| 2 | 1.56 | 1.22 - 2.00 | < 0.001 |
| 3 | 1.74 | 1.37 - 2.22 | < 0.001 |

**Table *S4.*** Multi-linear regression to estimate the association between schizophrenia with the COVID-19-related length of hospitalization among SARS-CoV-2-positive individuals

| Independent variables | Dependent variable | | |
| --- | --- | --- | --- |
|  | **Log transformed length of hospitalization** | | |
|  | **β (beta) coefficient** | **Std. Error** | **P-value** |
| (intercept) | -6.80 | 0.107 | <0.001 |
| Schizophrenia | 1.20 | 0.063 | <0.001 |
| Male | 0.08 | 0.038 | 0.025 |
| Sector (non-Haredi Jews) | **Ref** |  |  |
| Arab | 0.02 | 0.059 | 0.712 |
| Ultra-Orthodox Jews | -0.07 | 0.051 | 0.205 |
| SES (1-4) | Ref |  |  |
| 5-7 | -0.08 | 0.047 | 0.087 |
| 8-10 | -0.21 | 0.062 | <0.001 |
| Age (<50) | **Ref** |  |  |
| 50-59 | 0.16 | 0.046 | <0.001 |
| 60-69 | 0.29 | 0.058 | <0.001 |
| 70-79 | 0.67 | 0.081 | <0.001 |
| 80+ | 2.03 | 0.133 | <0.001 |
| BMI (kg/m^2^) | 0.01 | 0.017 | 0.737 |
| Smoking (yes vs no) | 0.22 | 0.046 | <0.001 |
| Comorbidities (0) | **Ref** |  |  |
| 1 | -0.02 | 0.044 | 0.588 |
| 2 | 0.15 | 0.057 | <0.001 |
| 3 | 0.27 | 0.059 | <0.001 |

**Table *S5*:** Multivariable logistic regression models showing the association between schizophrenia with all-cause 30-day and 1-year mortality among SARS-CoV-2 test-positive individuals, adjusting for sex, sector, SES, age, BMI, smoking, and the number of comorbidities

| Independent variables | Dependent variable | | | |
| --- | --- | --- | --- | --- |
|  | **30-day mortality** | | **One-year mortality** | |
|  | **OR (95%CI)** | **P-value** | **OR (95%CI)** | **P-value** |
| Schizophrenia | 9.07 (3.11 - 26.44) | < 0.001 | 6.27 (2.73-14.39) | <0.001 |
| Male | 2.74 (0.91- 8.30) | 0.073 | 2.45 (1.08 - 5.56) | 0.032 |
| Sector (non-Haredi Jews) | **Ref** |  |  |  |
| Arab | 2.57 (0.50 - 13.25) | 0.038 | 0.74 (0.19 - 2.94) | 0.623 |
| Ultra-Orthodox Jews | 3.97 (1.12-14.03) | 0.136 | 1.19 (0.45 - 3.15) | 0.505 |
| SES (1-4) | **Ref** |  |  |  |
| 5-7 | 0.97 (0.47 - 2.00) | 0.935 | 0.84 (0.51 - 1.38) | 0.667 |
| 8-10 | 0.51 (0.17 - 1.52) | 0.227 | 0.32 (0.14 - 0.73) | 0.723 |
| Age (<50) | **Ref** |  |  |  |
| 50-59 | 1.78 (0.15 - 20.63) | 0.646 | 3.67 (0.94 - 14.32) | 0.061 |
| 60-69 | 12.35 (1.94 - 78.50) | < 0.001 | 8.79 (2.32 - 33.33) | <0.001 |
| 70-79 | 33.26 (4.46 - 248.08) | < 0.001 | 13.04 (2.81 - 60.55) | <0.001 |
| 80+ | 390.50 (53.59 - 2845.69) | < 0.001 | 112.09 (27.07 - 464.06) | <0.001 |
| BMI (kg/m^2^) | 0.97 (0.89 - 1.05) | 0.001 | 0.92 (0.88 - 0.96) | <0.001 |
| Smoking (yes vs no) | 3.02 (0.7 - 13.00) | 0.145 | 2.18 (1.36 - 3.49) | 0.001 |
| Comorbidities (0) | **Ref** |  |  |  |
| 1 | 0.71 (0.09 - 5.63) | 0.745 | 0.89 (0.21 - 3.78) | 0.877 |
| 2 | 1.15 (0.14 - 9.34) | 0.899 | 1.38 (0.32 - 5.95) | 0.668 |
| 3 | 3.22 (0.54 - 19.09) | 0.196 | 3.19 (0.89 - 11.41) | 0.074 |

Table S6. Logistic regression and multiple linear regression for the association between schizophrenia and COVID-19 outcomes after adjusting for sex, age, sector, BMI, smoking, SES, the number of comorbidities, and vaccination among people diagnosed with COVID-19

| Independent variables | Dependent variables | | |
| --- | --- | --- | --- |
|  | **Hospitalization** | | |
|  | **OR** | **95%CI** | **P-value** |
| Schizophrenia | 3.80 | 2.70 - 5.34 | <0.001 |
| Vaccination 1 | 1.07 | 0.73 - 1.57 | 0.727 |
| Vaccination 2 | 0.72 | 0.47 - 1.10 | 0.129 |
| Vaccination 3 | 0.66 | 0.45 - 0.96 | 0.029 |
| Length of hospitalizations (log-transformed) | | | |
|  | **β- coefficient** | **Std. Error (SE)** | **p-value** |
| (Intercept) | -6.53 | 0.113 | <0.001 |
| Schizophrenia | 1.18 | 0.063 | <0.001 |
| Vaccination 1 | -0.18 | 0.077 | 0.023 |
| Vaccination 2 | -0.04 | 0.077 | 0.569 |
| Vaccination 3 | -0.27 | 0.051 | <0.001 |
| All-cause 30-day mortality | | | |
|  | **OR** | **95%CI** | **P-value** |
| Schizophrenia | 4.54 | 1.54 - 13.38 | 0.006 |
| Vaccination | 0.32 | 0.20 - 0.51 | <0.001 |
| All-cause one-year mortality | | | |
|  | **OR** | **95%CI** | **P-value** |
| Schizophrenia | 4.98 | 2.23 - 11.15 | <0.001 |
| Vaccination | 0.34 | 0.25 - 0.48 | <0.001 |

Note: The reference group is individuals who did not receive any vaccination.

Supplement A

ICD codes for schizophrenia

295.0 - Simple type schizophrenia

295.1 - Hebephrenic schizophrenia

295.2 - Catatonic schizophrenia

295.3 - Paranoid schizophrenia

295.4 - Acute schizophrenic episode

295.5 - Residual schizophrenia

295.6 - Schizophreniform disorder

295.7 - schizoaffective disorder, chronic type

295.8 - Other specified schizophrenia

295.9 - Unspecified schizophrenia

Figure S1


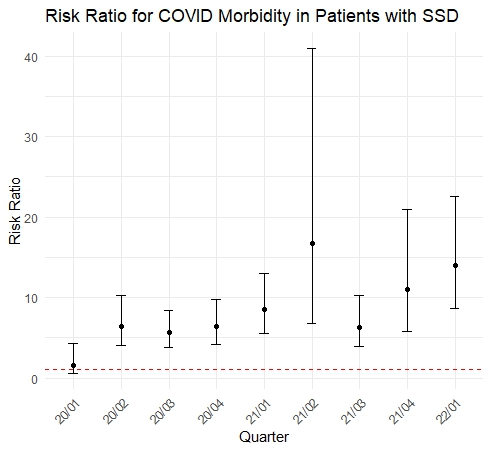


Risk ratio of severe COVID-19 among patients with schizophrenia spectrum disorders
